# Targeting Misconduct in Prison by Modifying Occupational Factors in Correctional Facilities

**DOI:** 10.3389/fpsyt.2020.00517

**Published:** 2020-06-05

**Authors:** Joanna Vogel, Julia Sauter, Bob-Oliver Vogel, Klaus-Peter Dahle

**Affiliations:** ^1^Department of Forensic Psychiatry, Charité - Universitätsmedizin Berlin, Berlin, Germany; ^2^Department of Psychiatry and Psychotherapy, Charité - Universitätsmedizin Berlin, Berlin, Germany; ^3^Department of Psychology, Universität Hildesheim, Hildesheim, Germany

**Keywords:** misconduct in prison, offender treatment, correctional officers, self-efficacy, sick days

## Abstract

Misconduct in prison is a phenomenon, which by its nature is hard to observe. Little is known about its origins and its modifiability. This study presents data on the level of misconduct in prison perceived by staff members and examines its impact on occupational factors. Data from officers, which also included i.e. team climate, job satisfaction, self-efficacy, and sick days, was collected at three different correctional units in Berlin, Germany (*N* = 60). The study reveals higher rates of perceived misconduct in prison on regular units as compared to treatment units within the observed facilities. In addition, regression analysis provides evidence for an association of rates of misconduct in prison, sick days, and low self-efficacy. Results are discussed in terms of providing a model that supports the idea of a network entailing occupational factors and misconduct in prison and which can be utilized to target misconduct in prison with suitable interventions.

## Introduction

Providing safety through regulation is one of the main aspects of the daily work of correctional officers. However, it is common for correctional facilities to be a place where both, inmates and officers, face highly adverse experiences. Adverse experiences can include a wide range of instances with varying degrees of violence, e.g. experiencing (directly) or witnessing (indirectly) physical assaults between inmates (1) or inmates and officers (2), sexual assault among inmates (3–6), or sexual harassment by forensic workers (7) to name only a few. For a complete overview of dangers that officers are confronted with, see Ferdik and Smith (8).

These and other adverse experiences at correctional facilities are harmful in many ways. Depending on the form of violence, inmates who are victimized are confronted with injuries and sexually transmitted diseases (9). In addition, the perception of an unsafe atmosphere as measured by a ward climate instrument is correlated to elevated levels of fear of self-disclosure, which can be regarded as an important aspect of therapy resistance (10). Furthermore, indirect or direct exposure to threats, violence, and the perception of not being safe in an environment can be harmful to inmates, employees, and visitors. A constantly growing body of research points to serious negative consequences in terms of job stress for employees of correctional facilities which are associated with the described adverse experiences (11). In comparison to other occupations, studies on prison officers also report elevated burnout rates (12–15), more frequently diagnosed post-traumatic stress disorder (16), and more drug use (17). These negative health outcomes do not only affect employees on a personal level but can also represent a burden for the organizations because of higher rates of absenteeism or job termination (18).

Negative experiences are made in prisons, especially when the rules intended to guarantee social order are not adhered to. Two models are used to explain the emergence of misconduct in prison. Criminal norm orientation and thus the activities were thought to either be brought into the institution by the inmates themselves (“importation model”; (19)), or the (criminal) subculture existing in the prison was regarded as the result of a process of adaptation to the depriving institutional factors (“deprivation model” (20, 21)). It has been argued that a very unique inmate code is formed within prisons, which the newcomers (must) join (21), and which is associated with very different forms of misconduct, including the negative outcomes mentioned above. Research has shown that both models are suitable to explain inmates’ misconduct (22, 23), which on the other hand shows that neither theory can be considered as complete (23).

One of the main purposes of a prison is to change inmates’ norm orientation while being incarcerated. This aim is not easily accomplished because these dissocial attitudes presumably existed before the imprisonment and led to imprisonment in the first place. It is likely that modifying inmates’ dissocial attitudes could also reduce the extent of misconduct. In the sense of the deprivation model, however, organizational and structural changes can be realized in an economic manner through policy adjustments, in order to reduce prison subculture. Feld (21) found that the more custodial and punitive settings, prison subculture was more violent, more hostile, and more oppositional than those in the treatment-oriented settings were. This is in line with recent evidence that emphasizes the role of prison overcrowding (24) and consequently, inmate-to-staff ratio. Gaining a deeper understanding of the occurrence and determinants of prison misconduct is the aim of this study. In doing so, possibilities of modifying the phenomenon in a way that makes correctional facilitates safer for both, inmates and officers, shall be explored.

### Research Questions

Previous research focused on the inmates’ perspective on misconduct in prison has shown that inmates perceive less misconduct on treatment units compared to regular prison units (25). Our group has previously published data on how occupational factors relate to prison’s social climate and treatment motivation of inmates (26). The studies are closely linked since they are both part of an evaluation project, overlapping of participants and psychometric measures will be described in detail in the following paragraphs. Now, in the present study we were aiming at addressing the following hypotheses focusing on prison misconduct: Firstly, we investigated whether officers also perceived differences in misconduct in prison on regular units compared to treatment units. Our hypothesis was that, as in inmates, differences should be perceived. Secondly, misconduct in prison being a fundamental part of the everyday experience of correctional officers, working on treatment units was hypothesized to correlate with occupational factors (OF) such as team climate, job satisfaction, self-efficacy, and sick days. These OFs have been studied and described before by our group (26). Thirdly, we hypothesized that OFs, especially the occurrence of sick days, predict the extent of prison misconduct on treatment units.

## Methods

The current study is part of an ongoing evaluation that started in 2014 and encompasses different correctional treatment programs in Berlin, Germany. The Ethics Committee of Charité - Universitätsmedizin Berlin approved of the study with a positive ethics vote (EA4/131/18).

Data was collected at social-therapeutic facilities for adult and adolescent offenders as well as on a preventive detention unit. In contrast to regular prison units, the aim of these therapeutic facilities is to establish a therapeutic community. In addition to psychotherapy, participants have access to targeted leisure activities and social work. This, together with a lower staff-inmate ratio, is intended to create a supportive climate in order to achieve the therapeutic goals, i.e. the reduction of recidivism (10, 26). The social-therapeutic facilities are not separate, but rather houses or even units within the regular prison. As a result of this, all the persons interviewed—officers as well as inmates—had spent some time on regular units before coming to the therapeutic facility.

### Participants

The acquisition of N = 60 participants was a two-step process. First, one third of all officers working at the social-therapeutic facility for male adult and adolescence offenders were randomly selected using the randomize function in Excel (Microsoft, Washington, USA). At the preventive detention unit quota sampling was used, resulting in a subsample that was proportional in terms of gender. All randomly selected participants gave their written consent and took part in an interview with a trained psychologist. In a second step also officers who were not chosen for an interview were able to volunteer and also gave their written consent (n = 12). An overall participation rate of 45% was observed across all sites (n = 42 male and n = 18 female). The participating officers work in treatment units most of the time. However, all of them have prior experience on regular units since it is part of their educational program. In addition, during their daily service it often occurs that officers are deducted from treatment units to regular units due to personal calamity. The subsample deriving from the social-therapeutic facility for male offenders consists of *n* = 20 correctional officers (33.3%; Age: *M* = 48.9 years; *SD* = 8.4; *Min-Max* = 34–59). In the social-therapeutic facility for adolescent offenders *n* = 15 correctional officers took part in the study (25.0%; Age: *M* = 47.4 years; *SD* = 8.4; *Min-Max* = 32–59). Twenty-five correctional officers (41.7%; Age *M* = 46.4 years; *SD* = 8.9; *Min-Max* = 30–57) from the preventive detention unit agreed to participate. Data from the same correctional officers studied in a previous paper (26) have been analyzed to investigate the influence of occupational factors on prison misconduct (previous study n = 63, current study n = 60).

### Procedure

Semi-structured interviews were conducted by psychologists (level of education: master’s degree or higher) within the institutions during working hours of the officers. Interviews took between 1.5 and 2 h and included, among others, different questionnaires (shortened and/or adopted from previous research and own developments) covering misconduct in prison, team climate, job satisfaction, and self-efficacy.

### Psychometric Measures

#### Interviews With the Participants About Subjectively Perceived Misconduct in Prison (PMP)

We decided to record self-reported misconduct. It can be assumed that misconduct that was not always considered as official could also be a burden for employees (e.g. hierarchies, verbal threats). Since it was precisely the individual effects of the employees that were the focus of the study, recording subjective perception seemed to be of crucial importance. As both self-reported and official misconduct had been valid and reliable indicators of inmate behavior in previous studies (23, 27), we felt that such an approach was appropriate. All officers were asked about the extent of misconduct they perceived using a Likert-scale (0 = never to 3 = often). A total of eleven questions cover very different forms of misconduct (e.g. drugs/alcohol, sexual assault; see **Table 1** for an overview). Nine of the questions focus on possible misconduct by inmates. The other two questions, on the other hand, focus on possible misconduct committed by prison staff (unjustified priority treatment and suppression). Each officer completed the questions for two work sites, i.e. regular and treatment units. Internal consistency was measured as Cronbach’s Alpha for the PMP as rated by officers for treatment units and regular units is acceptable (*ρ_T_* =.794; *ρ_T_* =.775). The PMP measures the perception of misconduct and is not an objective measure.

**Table 1:** Perceived Misconduct in Regular Units vs. Treatment Units from Officers’ Perspective (n = 60).

	*RU*	*TU*	*95% CI for Mean Difference*		*Paired Samples T- Test*			
	*M (SD)*	*M (SD)*	*Lower*	*Upper*	*t*	*df*	*p*	*Cohen’s d*
*Total*	2.2 (0.4)	1.6 (0.4)	−.69	−.46	−10.0	59	***	−2.60
*Hierarchies*	2.9 (0.3)	2.4 (0.7)	−.74	−.36	−5.9	59	***	−1.54
*Unjustified priority*	1.9 (0.6)	1.8 (0.6)	−.17	.04	−1.3	59	.209^ns^	−.39
*Being suppressed*	0.8 (0.8)	0.4 (0.6)	−.65	−.30	−5.4	59	***	−1.41
*Illegal transactions*	2.7 (0.6)	2.5 (0.7)	−.38	−.11	−3.8	59	***	−.99
*Drugs/alcohol*	2.7 (0.6)	2.4 (0.7)	−.63	−.29	−5.6	59	***	−1.46
*Physical conflicts*	2.4 (0.6)	1.6 (0.6)	−.96	−.64	−10.2	59	***	−2.66
*Blackmailing*	2.4 (0.6)	1.5 (0.7)	−1.05	−.66	−8.7	59	***	−2.27
*Verbal threats*	2.7 (0.5)	2.0 (0.7)	−.86	−.50	−7.6	59	***	−1.98
*Weapons*	1.6 (0.8)	1.0 (0.7)	−.83	−.43	−6.4	59	***	−1.66
*Payments*	2.2 (0.7)	1.5 (0.8)	−.91	−.49	−6.6	59	***	−1.72
*Sexual assault*	1.8 (0.8)	1.0 (0.7)	−1.07	−.62	−7.5	59	***	−1.96

***p < .001; ns, not significant.

RU, regular unit; TU, treatment unit; M, mean; SD, standard deviation; df, degrees of freedom.

#### Team Climate

The Team Climate Inventory (TCI; (28)) is a questionnaire aiming at measuring work atmosphere in teams. The initial 44-item TCI was shortened to 15 items for economic reasons of the evaluation project: The remaining items cover three subscales: (1) safety (5 items), (2) vision (7 items), and (3) task orientation (3 items). (1) Safety measures the environmental perception of safety and the possibility of participation in decision-making. (2) Vision captures the aim of a team. (3) Task orientation collects efforts of the team members to further develop performance and quality of work (Likert-scale: 1 = not at all to 5 = completely). Internal consistency for the shortened TCI is good (*ρ_T_* =.839).

#### Job Satisfaction

To gather data on job satisfaction, unpublished adaptions derived from the abridged Job Descriptive Index (JDI; (29)) and the SAZ (Skala zur Erfassung der Arbeitszufriedenheit; (30)) were used. The specifically tailored job satisfaction scale entails eight items asking about satisfaction with colleagues, supervisor, work task, working conditions, organization, management, workload, and opportunities (Likert-scale: 0 = completely unsatisfied to 5 = completely satisfied). Internal consistency for the adapted job satisfaction scale is good (*ρ_T_* =.817).

#### Self-Efficacy

Two unpublished versions (for teachers and nurses) of the general self-efficacy scale (SWE; (31)) were shortened and adopted for the use in correctional facilities. The five items of the questionnaire measure perceptions of self-efficacy of officers in dealing with difficult and suspicious inmates (Likert-scale: 1 = strongly disagree to 4 = strongly agree). Internal consistency for the adapted self-efficacy scale is questionable (*ρ_T_* =.651).

#### Sick Days

Data on sick leave were collected *via* the administrative council of the facilities. Due to data protection regulations sick days could only be collected as average numbers per year. The studied OFs, namely Team climate, Job satisfaction, Self-efficacy, and Sick days are also described in detail in our previous study (26).

### Statistical Analysis

Statistical analysis was performed with SPSS 25.0 for Mac OS (IBM, Armonk, NY). First, paired-samples (two-tailed) t-tests were conducted to test for differences in perceived misconduct in prison ratings between regular units and treatment unit. Next, Pearson-correlations were calculated for perceived misconduct in prison ratings on treatment units and OFs (team climate, job satisfaction, sick days, and self-efficacy). According to Cohen (32), values of 0.1 and above represent a small effect, 0.3 and above represent a moderate effect and 0.5 and above represent a strong effect. Bonferroni-corrections were applied to all tests. Lastly, perceived misconduct in prison ratings from treatment units, but not regular units, were linear, stepwise regressed on OFs. All variables were normally distributed (Kolmogorov-Smirnov-Test: p-value Range =.051–.689).

## Results

### Officers’ Perception of Misconduct in Prison in Regular and Treatment Units

Bonferroni-corrected t-tests revealed that correctional officers perceived overall more misconduct in prison in regular as compared to treatment units (see **Table 1**). In fact, that difference in perception holds for all subscales except *unjustified priority by staff members* (*p* =.209).

### Misconduct in Prison and Its Correlation With Team Climate, Job Satisfaction, and Sick Days

Two-tailed Pearson-correlations for perceived misconduct in prison ratings and OFs (team climate, job satisfaction, sick days, and self-efficacy) confirmed our hypothesis that perceived misconduct in prison moderately correlate with team climate (*r* = −.34, *p* < .05), job satisfaction (*r* = −.38, *p* < .01), and sick days (*r* =.42, *p* < .01).

**Figure 1:**
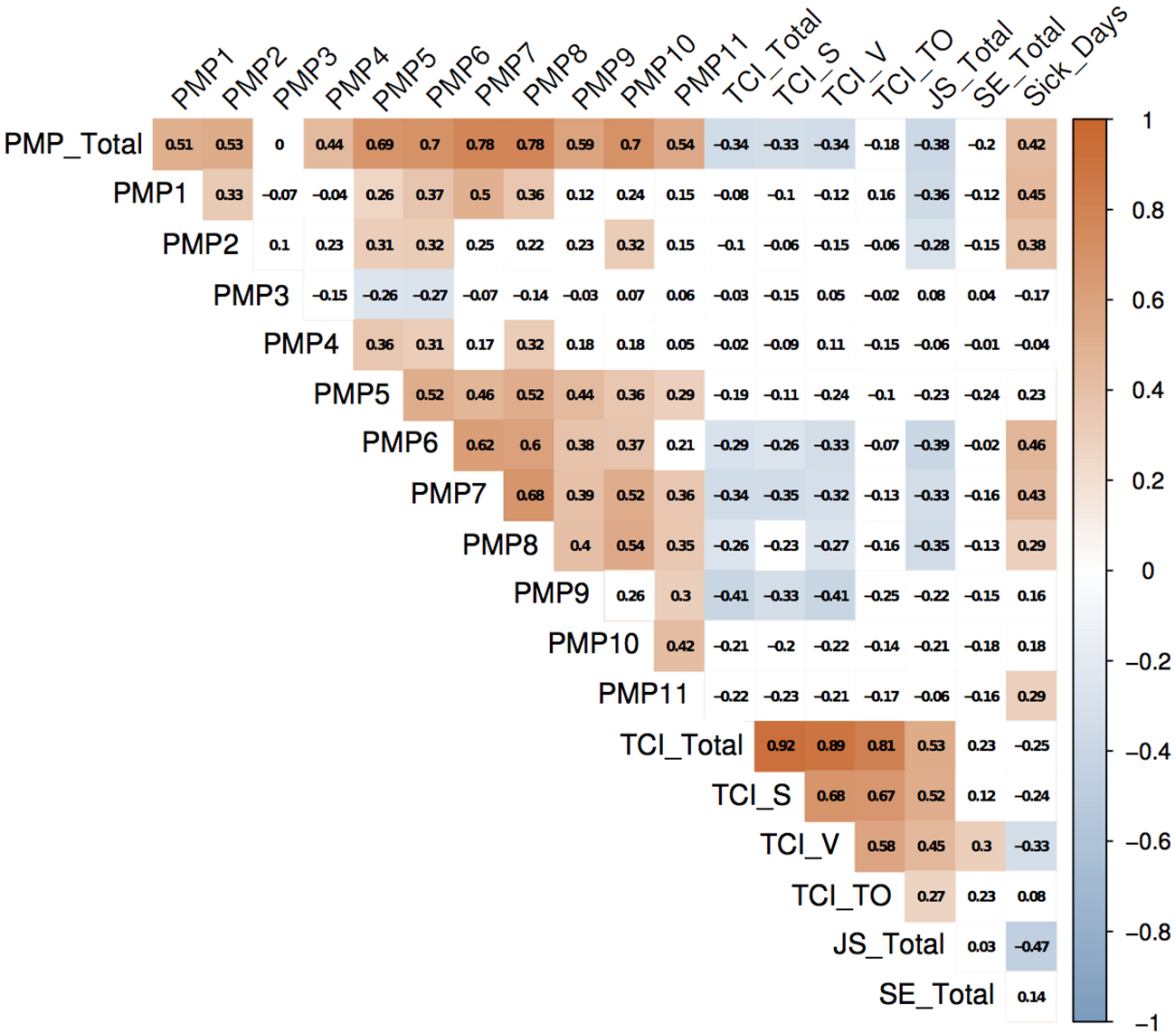
Correlation matrix of different aspects of perceived misconduct in prison and occupational factors. Significant correlations range from dark blue (+1) to dark red (−1). Insignificant correlations are shown in white. Each square is showing the correlation coefficient. JS, job satisfaction; PMP, perceived misconduct in prison; SE, self-efficacy; TCI, team climate inventory; TCI_S, team climate inventory subscale safety; TCI_V, team climate inventory subscale vision; TCI_TO, team climate inventory subscale task orientation.

Other than assumed, self-efficacy did not correlate significantly with perceived misconduct in prison in treatment units (*r* = −.20, *p* =.126). Having a closer look at the full correlation matrix (see **Figure 1**), it becomes apparent which types of perceived misconduct in prison are associated with which OF on treatment units. Team Climate is correlated negatively to physical conflicts between inmates (*r* = −.29, *p* < .05), blackmail (*r* = −.34, *p* < .01), verbal threats (*r* = −.26, *p* < .05), and the detention of (self-built) weapons (*r* = −.41, *p* < .01) on treatment units. Job satisfaction is correlated negatively to hierarchies between inmates (*r* = −.36, *p* < .01), unjustified priority treatment by staff members (*r* = −.28, *p* < .05), physical conflicts (*r* = −.39, *p* < .01), blackmail (*r* = −.33, *p* < .05), and verbal threats between inmates (*r* = −.35, *p* < .01).

Sick days are correlated positively to hierarchies (*r* =.45, *p* < .01), unjustified priority treatment by staff members (*r* =.38, *p* < .01), physical conflicts (*r* =.46, *p* < .01), blackmail (*r* =.43, *p* < .01), verbal threats (*r* =.29, *p* < .05), and sexual assaults between inmates (*r* =.29, *p* < .05). As the complete correlation matrix shows, OFs are also associated with each other. In particular, team climate is moderately correlated to job satisfaction (*r* =.53, *p* < .01) and job satisfaction is moderately correlated to sick days (*r* = −.47, *p* < .01).

### Influence of Sick Days and Self-Efficacy on Perceived Misconduct in Prison

Our third hypothesis, i.e. OFs predicting perceived misconduct in prison, was confirmed for treatment units (F(2,52) = 7.68, *p* < .001), and to be more specific for sick days (*R^2^* =.16) and self-efficacy (*R^2^* =.23, see **Table 2**). Job satisfaction (*β* = −.21, *p* =.134) and team climate (*β* = −.242, *p* =.065) were not significantly associated with perceived misconduct.

**Table 2:** Influence of Occupational Factors on Perceived Misconduct in Prison (n = 60).

	*B*	*SE*	*β*	*R^2^*
*Sick Days*	.009	.003	.430***	.160
*Self-Efficacy*	−.054	.025	−.262*	.228
*Job Satisfaction*	−.013	.014	−.211^ns^	
*Team Climate*	−.359	.312	−.242^ns^	

*p < .05; **p < .01; ***p < .001; ns, not significant.

## Discussion

This study investigated the relationship between misconduct in prison and occupational factors from the officers’ perspective. The results support the idea that OFs are associated with various forms of misconduct in treatment units that can corrupt safety and rehabilitation in correctional facilities. The results of the study also provide the possibility to speculate on hypothetical starting points for modifying prison misconduct in such a way that the experience of imprisonment and imprisoning might become safer, and thus comes closer to the legal goal of rehabilitation treatment.

Officers perceived less misconduct in prison on treatment units compared to regular units. This finding complements work from Sauter and colleagues (25), according to which the inmates also reported less misconduct in treatment units. The study has also shown that OFs are correlated not only to each other but also to different aspects of misconduct in prison on treatment units. Also, occupational factors, i.e. sick days and self-efficacy of officers together explain 22.8% of variance in misconduct in treatment units. The majority of studies investigating the risk factors of misconduct in prison have focused on inmate’s characteristics such as sex and prior record (23) as well as prison characteristics such as prison crowding (33, 34). In a meta-analysis French & Gendreau (35) have identified three main strategies that can be utilized to effectively lower prison misconduct: a) “get tough” meaning very low levels of service and strategies such as solitary confinement in order to discipline inmates, b) “situational control strategies” including variables such as prison climate and inmate-to-staff-ratio, and c) treatment programs that aim at behavioral changes of the inmates. The highest effectiveness was found for behavioral treatment programs (r =.26). Fewer studies have investigated how factors related to correctional officers influence prison misconduct of inmates. Recently, several studies have highlighted the importance of staff-related factors in relation to inmate’s behavior. Findings from 3,886 inmates in Ohio (USA) prisons suggest that inmates’ perceived legitimacy of correctional officers results in fewer nonviolent infractions (36). However, perceived legitimacy was not associated with violent misconduct in this study (36). Moreover, Logan and colleagues (37) highlight the importance of the staff-inmate relationship and state that officers can affect inmates’ behavior positively and negatively during their incarceration (e.g. (38, 39)). Taken together, these studies highlight the importance of investigating factors related to correctional officers in order to influence inmate’s behavior, including misconduct. Our results provide first time evidence that self-efficacy and sick days of correctional officers are related to perceived misconduct in prison, therefore highlighting the potential of these factors in reducing prison misconduct.

By its design, the study provides us with the possibility of speculating on starting points to create interventions in order to target the extent of harmful misconduct in prison. The study shows that misconduct in prison is associated with sick days. Important to note, sick leave itself can be a result of exposure to violence and threat at work (40, 41). Combining these results suggests a possible network of adverse experiences in prison that is further supported by the correlations found in the study.

The proposed network provides us with multiple hypothetical starting points for planning interventions in order to reduce destructive misconduct in prison. One target point could be to limit the potentially cost intensive consequences of sick leave by temporarily providing competent employee replacement. Even better, personal levels could be increased all together. In that way, well-educated and accustomed staff could serve as a suitable replacement for colleagues in sick leave right away. Another targeting point could be to create programs to elevate levels of team climate, job satisfaction, and self-efficacy. An improvement of team climate could be achieved by implementing team supervisions and team building. Job satisfaction could be improved by shaping working conditions, organization, management, and workload. Improving OFs, especially sick days, might result in less misconduct, fewer incidence of exposure to adverse and violent experiences and therefore levels of OFs should increase. The following limitations should be considered: Data presented here relies solely on the officers’ perception of misconduct in prison. However, using the same questions Sauter and colleagues (25) found that perceptions on misconduct did not differ in the overall picture between inmates and officers. Only minor differences were found, most likely due to distortions caused by in- and out-group biases (42, 43). Another important limiting factor is that the results represent solely association findings which do not imply causality or a direction of effect. Therefore, the discussed network, as well as the proposed interventions are partly hypothetical and need further research in order to gain more insight into possible causal effects. Also, the sample size is low and complementing data from officers working in regular units most of their daily routine should be collected to further investigate the relationship of occupational factors and misconduct in regular prison units. Another limitation is that the adapted and revised questionnaires which derived from already existing and validated questionnaires are so far not validated. The reason for modifying the questionnaires was to make the interviews as economical as possible. The questionable reliability of the self-efficacy scale has to be emphasized at this point. It is possible that the instrument was not adapted in a suitable manner and decreased in item number too drastically. Further research on this instrument is needed.

The aim of this study was to provide empirical data on the potential of occupational factors to help to create an atmosphere that can prevent or at least minimize misconduct in prison. Officers’ care therefore not only seems to pay off for the officers themselves, but also seems to be suitable for coming closer to the legal goal of rehabilitation and resocialization. Sick days and self-efficacy were identified as being linked to misconduct in prison and thereby added to a growing body on literature on misconduct and its correlates in correctional facilities. The paper presented hypothetical interventions that might influence the extent of misconduct. Longitudinal future studies have to investigate if and under what circumstances misconduct can be minimized with the proposed interventions.

## Disclaimer

The views expressed are those of the authors and not necessarily those of the Senate of Justice and Consumer Protection of Berlin.

## Data Availability Statement

The datasets generated for this study will not be made publicly available. This study is part of an evaluation project commissioned by the Berlin Senate for Justice, Consumer Protection and Anti-Discrimination. We do not have the right to disclose the data.

## Ethics Statement

The studies involving human participants were reviewed and approved by ethics committee of Charité - Universitätsmedizin Berlin. The patients/participants provided their written informed consent to participate in this study.

## Author Contributions

JV: data analysis and preparation and revision of manuscript. JS: questionnaire design, manuscript preparation and revision. B-OV: manuscript preparation and revision, figures. K-PD: study supervision, administrative, technical, and material support, manuscript revision.

## Funding

The Senate of Justice and Consumer Protection of Berlin, Germany funded the evaluation project.

## Conflict of Interest

The authors declare that the research was conducted in the absence of any commercial or financial relationships that could be construed as a potential conflict of interest.
